# Dual Oxytocin Signals in Striatal Astrocytes

**DOI:** 10.3390/biom15081122

**Published:** 2025-08-04

**Authors:** Elisa Farsetti, Sarah Amato, Monica Averna, Diego Guidolin, Marco Pedrazzi, Guido Maura, Luigi Francesco Agnati, Chiara Cervetto, Manuela Marcoli

**Affiliations:** 1 Department of Pharmacy, University of Genova, Viale Cembrano 4, 16148 Genova, Italy; 2 Department of Experimental Medicine, University of Genova, Viale Benedetto XV 1, 16132 Genova, Italy; 3 Department of Neuroscience, University of Padova, Via Gabelli 63, 35122 Padova, Italy; 4 Department of Earth, Environment and Life Sciences, University of Genova, Viale Benedetto XV 5, 16132 Genova, Italy; 5 Department of Biomedical, Metabolic Sciences and Neuroscience, University of Modena and Reggio Emilia, 41121 Modena, Italy; 6 IRCCS Ospedale Policlinico San Martino, 16132 Genova, Italy; 7 Interuniversity Center for the Promotion of the 3Rs Principles in Teaching and Research (Centro 3R), 56122 Pisa, Italy

**Keywords:** oxytocin receptor, astrocyte processes, glutamate release, intracellular calcium, biased agonists, oxytocin, atosiban, carbetocin

## Abstract

The ability of the neuropeptide oxytocin to affect glial cell function is receiving increasing attention. We previously reported that oxytocin at a low nanomolar concentration could inhibit both astrocytic Ca^2+^ signals and glutamate release. Here, we investigate the ability of oxytocin receptors to couple both inhibitory and stimulatory pathways in astrocytes, as already reported in neurons. We assessed the effects of oxytocin at concentrations ranging from low to high in the nanomolar range on intracellular Ca^2+^ signals and on the glutamate release in astrocyte processes freshly prepared from the striatum of adult rats. Our main findings are as follows: oxytocin could induce dual responses in astrocyte processes, namely the inhibition and facilitation of both Ca^2+^ signals and glutamate release; the inhibitory and the facilitatory response appeared dependent on activation of the G_i_ and the G_q_ pathway, respectively; both inhibitory and facilitatory responses were evoked at the same nanomolar oxytocin concentrations; and the biased agonists atosiban and carbetocin could duplicate oxytocin’s inhibitory and facilitatory response, respectively. In conclusion, due to the coupling of striatal astrocytic oxytocin receptors to different transduction pathways and the dual effects on Ca^2+^ signals and glutamate release, oxytocin could also play a crucial role in neuron–astrocyte bi-directional communication through a subtle regulation of striatal glutamatergic synapses. Therefore, astrocytic oxytocin receptors may offer pharmacological targets to regulate glutamatergic striatal transmission, which is potentially useful in neuropsychiatric disorders and in neurodegenerative diseases.

## 1. Introduction

It is a recognized fact that astrocytic–neuronal bidirectional communication is essential to maintain brain functions. Astrocytes express GPCRs and ligand–gated ion channels that can be acted upon by various neurotransmitters (see [1,2,3,4]) and can release gliotransmitters (among which the best-known is glutamate) that can act upon neuronal receptors [5]. In particular, astrocytes are involved in the subtle regulation of glutamatergic synapse transmission [6,7,8]. Indeed, perisynaptic astrocyte processes (PAPs) at tripartite glutamatergic synapses can modulate synaptic transmission and neuronal plasticity [6,9,10], release glutamate (see [11,12,13,14,15]), and tune synapse coverage, therefore regulating the extracellular space volume [16] and glutamate clearance. In the striatum, astrocytes seem involved in emotion, cognition, and sensory processing and in substance abuse disorders [17,18]. Striatal astrocytes express dopamine receptors, and when dopamine is released by neighboring dopaminergic neurons, modification in intracellular calcium levels could be observed in astrocytes [19]. These cells are then implicated in modulating (inhibiting) dopamine transmission at the striatal synapse [20,21,22]. Indeed, this bidirectional interaction suggests a role of astrocytes in fine-tune dopamine signaling, crucial for emotional regulation, motivation, and decision-making [23]. Moreover, the involvement of astrocytic Ca^2+^ in the striatum has been related to repetitive grooming behaviors in rodents, suggesting the role of striatal astrocytes in mental health disorders as well [24]. Astrocyte dysfunction and altered control of striatal glutamatergic transmission have emerged in schizophrenia [25,26,27]; changes at striatal glutamatergic synapses, with ultrastructural expansion of PAPs and altered regulation of glutamate transmission, followed by augmented release of glutamate and spillover into the extracellular space, have been suggested as factors in the pathophysiology of Parkinson’s disease (PD) [28]. The striatum is a highly integrative brain area and receives glutamatergic inputs from the cortex and thalamus [29,30] targeting GABAergic medium spiny neurons. These inputs are crucial for synaptic development, during the early postnatal period, and for activity-dependent plasticity, including long-term potentiation and depression, processes at the basis of learning and memory (see [31]).

In glutamate transmission in adult striatal neuron–astrocyte networks, oxytocin (OT) and its receptors (OTRs) could play a significant role [32,33,34]. OT, a neuropeptide mainly produced in hypothalamic neurons, is involved in complex brain functions, from the modulation of motor pathways [35] to the approach to novelty [36] and social recognition [37], as well as behaviors and emotionality that support reproduction and social interactions [38]. Moreover, OT plays roles in cognitive and emotional functions, acting not only as a neurotransmitter but also as a hormone, with complex mechanisms. In fact, OT is involved in bonding and orgasm [39] and in reproduction, with mechanisms that are not only centrally mediated [40,41]. OT mediates its central effects by acting upon widespread distributed OTRs in brain regions including areas involved in neuropsychiatric disorders or neurodegenerative diseases such as depression, anxiety, schizophrenia, autism, attention deficit hyperactivity disorder, PD, and Alzheimer’s disease [42]. A mismatch has been reported between projections of OT neurons and OTR expression in the brain, suggesting indirect non-synaptic OT signaling [43] and thus an action through volume transmission [44]. OTRs are found in several brain regions; among them, some are crucial for social behavior, emotional regulation, and stress response, and their dysregulation is implicated in various neuropsychiatric disorders (such as autism spectrum disorders, attention deficit/hyperactivity disorder, schizophrenia, post-traumatic stress disorder, anxiety, and depression). The amygdala, hypothalamus, hippocampus, nucleus accumbens, and prefrontal cortex are some of these areas [45]. The actions of OT in the brain have been mainly investigated in neuronal cells [46,47,48], and the ability of OT to affect the responses of glial cells has long been neglected. However, recent evidence supports the ability of OT to also have an impact on astrocyte functioning. In particular, it has been reported that OT triggers astrocyte Ca^2+^ transients [49,50,51,52] and induces rapid and reversible astrocytic morphological changes [53], mediating modulation in neuro-astrocyte network activity, and that OTR signaling in astrocytes contributes to anxiolysis and positive reinforcement [52,54]. Also, we have reported that OTRs are expressed on astrocyte processes in adult rat striatum, namely in fine PAPs that are primarily involved in the communication between neurons and astrocytes at tripartite synapses. The activation of these OTRs was found to inhibit the evoked Ca^2+^ signals and release of glutamate from the processes, suggesting that astrocytic OTR can regulate the glutamate transmission in adult striatal neuron–astrocyte networks [32,33,34]. Quite interestingly, the inhibitory effect on the Ca^2+^ signals and glutamate release from the processes was observed at a nanomolar concentration of OT (3 nM), while at lower or higher concentrations OT was ineffective. This atypical concentration–response curve replicated previous observations on striatal neurons. As demonstrated by Romero-Fernandez et al. [55], the same 3 nM OT concentration was found to evoke inhibitory effects at striatal neurons, while lower or higher concentrations proved to be ineffective. This atypical OT concentration–response curve and the narrow concentration dependency of the OT effect are somewhat puzzling phenomena.

It is noteworthy that OTRs were reported as being able to couple to both inhibitory and stimulatory pathways in neuronal cells [56,57,58]. Excitatory OT actions through OTRs coupled to G_q_ and IP3 signaling have been repeatedly observed, with increased intracellular Ca^2+^ and neuronal excitation (see [59,60,61]). On the other hand, evidence is accumulating that OT can also act through inhibitory intracellular pathways in neurons. Indeed, presynaptic OTRs were reported to inhibit the neurotransmitter release (glutamate or GABA) by modulating the Ca^2+^ entry into the terminals [62,63,64]. Notably, OTR ligands were identified that behave as a “biased agonist,” selectively activating the inhibitory G_i_ or the excitatory G_q_ pathway [65,66].

Here, to better understand the narrow concentration dependency of the OT effect on the processes of adult striatal astrocytes, the ability of OT to couple to both the inhibitory and the stimulatory pathways in astrocyte processes was assessed. We evaluated the effects of OT at concentrations ranging from a low to high nanomolar range by investigating the release of the gliotransmitter glutamate and on the intracellular [Ca^2+^] signals in the processes of adult rat striatal astrocytes, in addition to checking the transduction pathways involved. Furthermore, we investigated the effect of the biased agonists atosiban and carbetocin, which preferentially activate the inhibitory and stimulatory signaling pathways, respectively, on intracellular [Ca^2+^] signals and glutamate release in the processes.

## 2. Materials and Methods

### 2.1. Animals

Striatal tissues were collected according to [67,68], from adult male Sprague–Dawley rats (200–250 g). These animals were raised and housed in the animal facility of the Department of Pharmacy (DIFAR), University of Genova, Italy. Temperature (22 ± 1 °C), relative humidity (50%), and light period during the day (12 h light/dark cycle, with light from 7 a.m. to 7 p.m.) were constantly controlled. Standard diet and water ad libitum were guaranteed to the rats. The animal care complied with the European Communities Parliament and Council Directive 2010/63/EU and with the Italian D.L. n° 26/2014. Animal use was approved by the Italian Ministry of Health (protocol number 75F11.N.0RF of 16 November 2021), in accordance with Decreto Ministeriale 116/1992. We adhered to the 3R principles to minimize the number of rats used and distress caused.

### 2.2. Preparation of Purified Striatal Astrocytic Processes

Immediately following the collection of striatum, the tissue was immersed in a buffered sucrose solution (0.32 M sucrose, 10 mM Tris/HCl; pH 7.4) that had been cooled in ice. Purified astrocyte processes (gliosomes) were prepared in accordance with the protocol developed by Nakamura [69]. The tissue was homogenized in the sucrose solution. When the experimental protocol required the presence of PTX, the toxin was added to the buffered sucrose solution at a concentration of 5 nM to entrap this agent into subsequently isolated gliosomes [70,71,72].

After tissue homogenization, the homogenate was subjected to rapid centrifugation to remove any debris and nuclei. On a discontinuous Percoll gradient (2, 6, 10, and 20% (*v*/*v*) in Tris-buffered sucrose), we gently stratified the supernatant. The gradient was centrifugated, and gliosomes were collected at the interface between the 2–6% Percoll solution. To completely remove Percoll and sucrose, the gliosomes were resuspended in a final HEPES standard medium and subjected to final centrifugation. The HEPES medium (pH 7.4) had the following composition (mM): NaCl 128, KCl 2.4, MgSO_4_ 1.2, KH_2_PO_4_ 1.2, CaCl_2_ 1.0, and HEPES 10 with glucose 10. Gliosomes were previously characterized as a purified preparation of astrocytic processes, negligibly contaminated by neuronal subcellular particles as demonstrated by an immunofluorescent assay and Western blot analysis [72,73,74]. Moreover, we previously observed that striatal gliosomes express GFAP, an astrocytic marker, and ezrin, a selective protein restricted to the perisynaptic process [75]. Furthermore, gliosomes were found to be positive for VGLUT1, one of the vesicular glutamate transporters that occur when glutamate is released in a Ca^2+^-dependent manner [71].

### 2.3. Endogenous Glutamate Release

To study the release of the endogenous glutamate, we applied the superfusion technique to a gliosomal monolayer in a continuous superfusion system with the HEPES medium maintained at 37 °C. Gliosomes were first monolayered at the bottom of the superfusion chambers, and then they were superfused at the rate of 0.5 mL/min for 33 min to stabilize the basal glutamate outflow. The fresh HEPES medium was replaced every 10 min. Superfusion avoids the creation of a receptor biophase and released substances have any indirect effect on neighboring gliosomes [32,33,34,71]. According to the experimental design, pharmacological tools (agonists and antagonists) could be added to the medium during superfusion to characterize the target’s pharmacological profile. This approach enables the investigation of the intracellular pathway and the exploration of potential interactions among pharmacological targets [76]. From t = 33 min, we collected 3 min samples (B1–B5) for each chamber superfusion; to evaluate the basal outflow of glutamate, we calculated the mean of the gliotransmitter concentrations in the first two fractions. At t = 38 min, gliosomes were exposed for 6 min to the depolarized stimulus (4-AP; 300 µM), alone or with OT at the concentration shown in the figures. The effect of the OT antagonist was assessed exposing gliosomes to L 371,257 8 min before OT. To investigate the role of phospholipase PLC in OTR-mediated effects, the inhibitor U73122 was added during superfusion 8 min before OT and 4-AP. To assess glutamate overflow during the experiment in the presence of a simple medium or a medium supplemented with an antagonist/inhibitor, in each experiment we superfused at least one chamber with a physiological medium or with a medium supplemented with an antagonist or other pharmacological tools. The overflows measured in these chambers were used as controls.

At the end of the superfusion experiments, on each gliosomal preparation we assessed protein quantification [77], while the glutamate released in the collected fractions was measured by inverse HPLC analysis [78]. For each superfusion fraction, glutamate was expressed as pmol/mg protein. The gliosomes superfused with the physiological medium were considered the control condition. Gliosomes superfused with the medium supplemented with L 371,257, U73122 or gliosomes with PTX entrapped during their preparation were considered the control for all the conditions in which these substances were used. To evaluate the effect of each pharmacological tool, the overflow of glutamate efflux in the control was subtracted from the overflow measured in drug-present conditions. For each experiment, the effect of OT, at the different concentrations, alone or in the presence of U73122 or PTX, was calculated as the % variation in the corresponding 4-AP alone or in the presence of the substances. The mean values of the all 4-AP overflows (as pmol/mg) were taken as 100%.

### 2.4. Intracellular [Ca^2+^] Assay

We assessed the cytosolic gliosomal [Ca^2+^]_i_ as previously described [33,34,79]. Briefly, gliosomes were washed with a physiological medium and incubated with Calcium Green™-1 AM (CG) at the used concentration of 10 μM for 30 min at 37 °C. Gliosomes were washed in the medium, transferred into black 96-well plates (50 μg/well), and then exposed to the indicated drugs. Using the top reading mode of the LB940 Mithras Fluorescence Multi-Label Reader (Berthold Technologies, Baden Württemberg, Germany), fluorescence intensities (excitation 485 nm, emission 535 nm) were measured every 10 s for 5 min. For each recording, the fluorescence value measured at the start time of the experiment was subtracted. At each recording time, we calculated the [Ca^2+^]_i_ variation (Delta Fluorescence) as the difference between the CG fluorescence recorded from the stimulated gliosomes and the value corresponding to the medium-treated gliosomes. For each experimental condition, we used the time courses of the [Ca^2+^]_i_ variations obtained in the medium and in stimulated conditions to calculate the Area Under the Curve (AUC).

### 2.5. Calculations and Statistical Analysis

In the figure legends, we report the means ± SEM of the number of experiments (*n*). To analyze the significance of the difference, we performed a *t* test, a one-way or two-way ANOVA, and Bonferroni’s post hoc test. We considered the data as statistically significant when *p* < 0.05. The t or F values are indicated in the legends. To perform statistical analysis, we used the Prism 4.02 software package (GraphPad Software, San Diego, CA, USA).

### 2.6. Materials

4-Aminopyridine (4-AP), U73122, Pertussis Toxin (PTX), atosiban, and oxytocin (OT) were purchased from Sigma-Aldrich (Milan, Italy), while L 371,257 and carbetocin were obtained from Tocris (distributed in Italy Bio-Techne SRL, Milan, Italy). The pharmacological tools were solubilized in distilled water. Salts were purchased from VWR, while Calcium Green™-1 AM was obtained from Life Technologies Italia (Milan, Italy).

## 3. Results

### 3.1. Release of Glutamate from Astrocyte Processes: Concentration-Dependent Responses to OT

**Figure 1:**
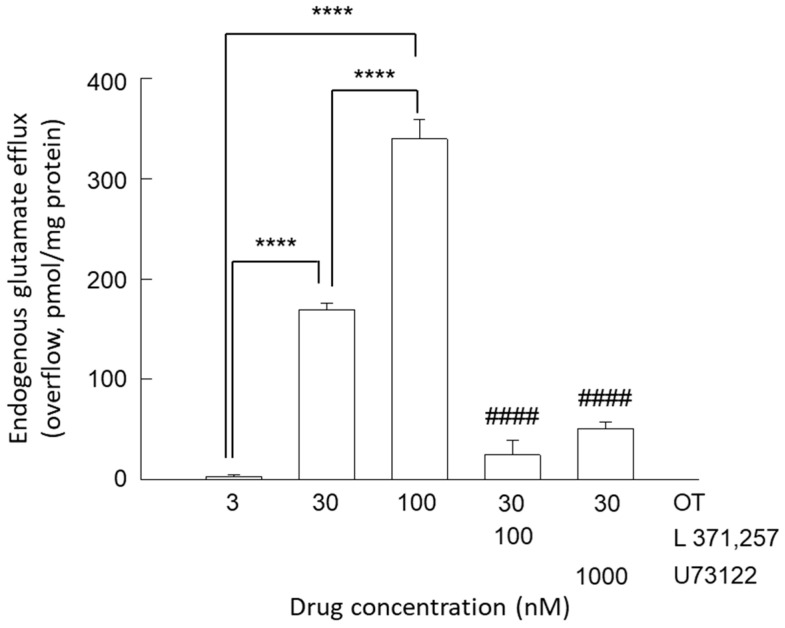
Endogenous glutamate efflux in response to OT in striatal gliosomes. Ineffectiveness of OT 3 nM and stimulation of the glutamate release by OT 30 nM and 100 nM. Antagonism by the selective OTR antagonist L 371,257 and the phospholipase PLC inhibitor U73122 of the release evoked by exposure to OT 30 nM. Bars represent the overflow of the endogenous glutamate efflux, expressed as pmol/mg of protein, in the presence of the drugs used at the concentrations indicated in the figure. Briefly, OT was added during superfusion (6 min) while the OTR antagonist (L 371,257 0.1 µM) and the PLC inhibitor (U73122 1 µM) were added 8 min before OT. In Section 2, further experimental details are reported. Data are the mean ± SEM of *n* = 5–7 independent experiments. One-way ANOVA analysis was applied to evaluate the effects of OT at different concentrations in the basal condition (*p* < 0.0001; F (16) = 430.9) and to assess the differences between the following groups: OT 30 nM, OT 30 nM + L371,257; OT 30 nM + U73122 (*p* < 0.0001; F (19) = 64.37). **** *p* < 0.0001 compared with the effect of OT 3 nM; #### *p* < 0.0001 compared with the effect of OT 30 nM according to Bonferroni’s post hoc test. OT, oxytocin.

Superfusion with a physiological standard medium of striatal gliosomes induced an efflux of the gliotransmitter glutamate equal to 78.77 ± 3.22 pmol/mg protein min (*n* = 54) as the mean value of the first two fractions collected. We found that while OT 3 nM was ineffective on release in resting conditions, confirming our previous data [32], at higher concentrations (30 and 100 nM) OT was able to evoke a glutamate efflux (Figure 1). Considering the observed effects, on OT 30 nM we assessed the selective OTR antagonist, L 371,257 (0.1 µM); the antagonist nullified the glutamate release evoked by OT (Figure 1), indicating that the OT-evoked glutamate release was dependent on OTR activation. Interestingly, the PLC inhibitor U73122 significantly inhibited the glutamate-releasing effect of OT, suggesting the involvement of the G_q_-PLC pathway.

**Figure 2:**
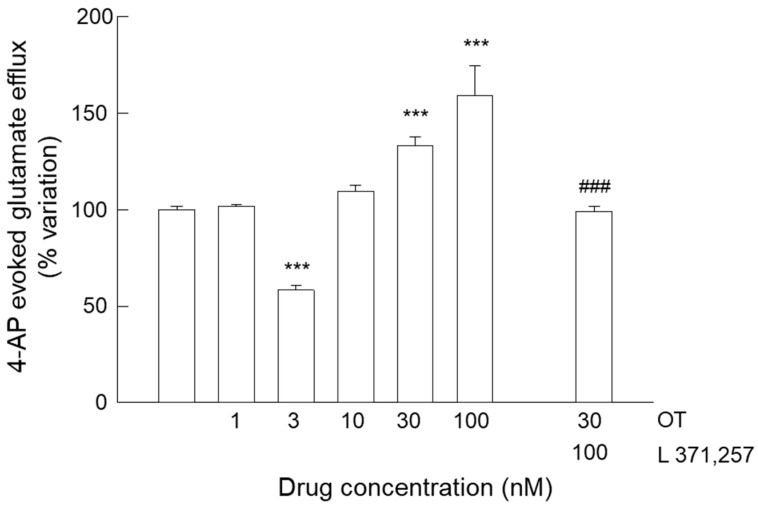
Endogenous glutamate release in response to 4-AP-induced depolarization in striatal gliosomes. Inhibitory effect of OT 3 nM and facilitatory effect of OT 30 nM and 100 nM on the 4-AP-evoked endogenous glutamate release; antagonism of the facilitatory effect of OT 30 nM by the OTR antagonist L 371,257. Bars represent the percent variation in the glutamate overflow due to 4-AP stimulation during superfusion and in the presence of the indicated drugs at the concentrations used. Briefly, 4-AP was added (6 min) during superfusion; OT was added together with 4-AP, while L 371,257 was added 8 min before the agonist. In Section 2, additional experimental details are reported. Data are expressed as mean ± SEM of *n* = 5–34 independent experiments. One-way ANOVA analysis was applied to evaluate the effects of OT at different concentrations in the 4-AP condition (*p* < 0.0001; F (92) = 121.4). *** *p* < 0.001 compared with the effect of 4-AP by Bonferroni’s post hoc test; a two-tailed *t* test was applied to evaluate the effects of the antagonist (t (22) = 5.575). ### *p* < 0.001 compared with the effect of 4-AP + OT 30 nM according to the two-tailed *t* test; 4-AP, 4-aminopyridine; OT, oxytocin.

As previously described by Amato and co-workers [39,40,41], OTR activation induces a significant reduction in the endogenous glutamate release when it was evoked by 4-aminopyridine (4-AP). The stimulation with 4-AP (300 µM) resulted in an augmented glutamate efflux, with the evoked overflow measuring 263.48 ± 4.58 pmol/mg protein; *n* = 34 (Figure 2). OT confirmed its ability to modulate the glutamate release evoked by quasi-physiological stimulus (4-AP) during superfusion. Activation of OTR by 3 nM OT induced robust inhibition of the 4-AP-evoked glutamate release, thereby confirming the capacity of OTR to regulate the gliotransmitter efflux. We confirmed the ineffectiveness of OT 10 nM in the modulation of the 4-AP-evoked glutamate efflux (see [39]), while at higher concentrations (30 and 100 nM) OT was able to increase the evoked release. As observed in the basal condition, the selective OTR antagonist L 371,257 completely abolished the overflow due to OT 30 nM (Figure 2).

Taken together, the findings suggest that OTR activation controls the glutamate release in striatal astrocyte processes, being able to evoke both inhibitory and facilitatory effects. In particular, at a concentration of 3 nM, only the inhibitory OT effect was observed on the evoked release, while at higher concentrations (30 and 100 nM), OT could facilitate both the resting and the evoked release.

### 3.2. Ca^2+^ Signals in Astrocyte Processes: Responses to OT

Previously, we reported that in the astrocytic processes the OTR activation by 3 nM OT significantly decreased Ca^2+^ signals in response to 4-AP [32,33].

**Figure 3:**
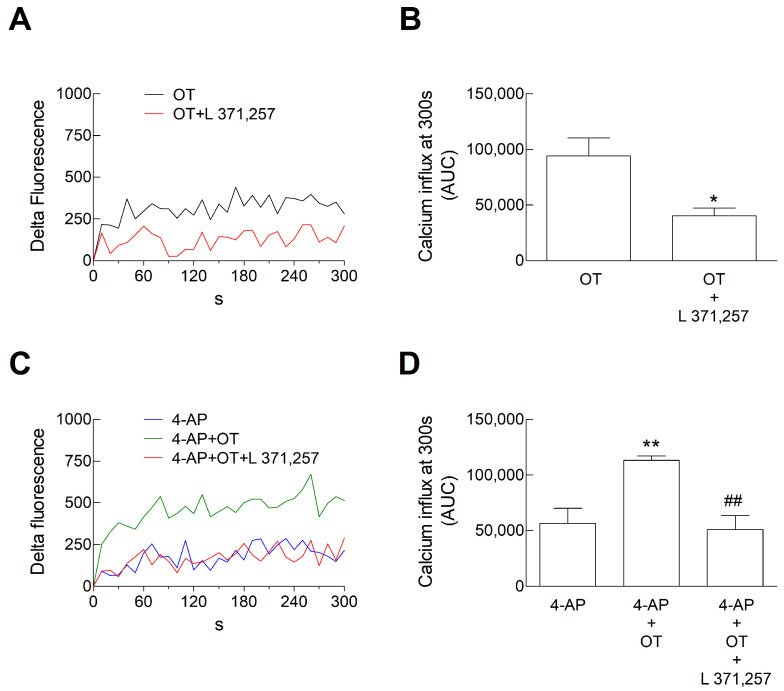
Calcium influx in response to 4-AP in striatal gliosomes. Gliosomes were loaded with CG and exposed to the indicated stimuli for 300 s at 37 °C. (**A**–**D**), and fluorescence related to CG was monitored every 10 s from 0 to 300 s. We calculated the “Delta Fluorescence” as [Ca^2+^]_i_ increase. Lines represent the mean values obtained from *n* = 5 independent experiments (**A**,**C**). The Ca^2+^ influx after 300 s was estimated by calculating the Areas Underlying the Curves (AUCs) and is reported in (**B**–**D**) for each experimental condition. Data are means ± SEM of *n* = 5 independent experiments. * *p* < 0.05 compared with the effect of OT 30 nM, according to the two-tailed *t* test (t (8) = 3.057) (**B**); ** *p* < 0.01 compared with the effect of 4-AP, while ## *p* < 0.01 compared with the effect of 4-AP in the presence of OT 30 nM, according to one-way ANOVA (*p* = 0.0028; F (14) = 10.03), followed by Bonferroni’s post hoc test (**D**). 4-AP, 4-aminopyridine; CG, Calcium Green™-1 AM; OT, oxytocin.

We here more extensively evaluate the OTR activation effects on the Ca^2+^ signals focusing on OT 30 nM. At this concentration, OT per se was able to increase the intracellular Ca^2+^ levels and the Ca^2+^ levels evoked by 4-AP in striatal processes (Figure 3). The selective OTR antagonist, L 371,257 (0.01 µM), significantly inhibited the facilitatory effect of OT (30 nM) on Ca^2+^ signals both in resting conditions (Figure 3A,B) and when the signal was evoked by 4-AP (Figure 3C,D), indicating that OT effects were mediated by OTR activation.

**Figure 4:**
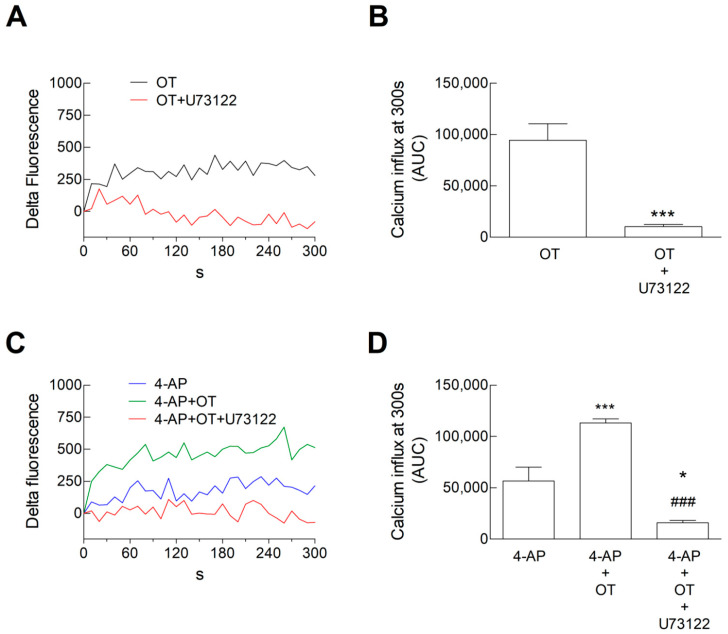
Calcium influx in response to 4-AP in striatal gliosomes. Gliosomes were loaded with CG and exposed to the indicated stimuli for 300 s at 37 °C. (**A**–**D**), and fluorescence related to CG was measured every 10 s from 0 to 300 s. We calculated the “Delta Fluorescence” as [Ca^2+^]_i_ increase. Lines represent the mean values from *n* = 5 independent experiments (**A**,**C**). The Ca^2+^ influx after 300 s was estimated by calculating the Areas Underlying the Curves (AUCs) and is reported in (**B**,**D**) for each experimental condition. *** *p* < 0.001 compared with the effect of 4-AP + OT 30 nM, according to the two-tailed *t* test (t (8) = 5.157) (**B**); * *p* < 0.05 or *** *p* < 0.001 compared with the effect of 4-AP; while ### *p* < 0.001 compared with the effect of 4-AP in the presence of OT 30 nM, according to one-way ANOVA (*p* < 0.0001; F (14) = 35.92), followed by Bonferroni’s post hoc test (**D**). 4-AP, 4-aminopyridine; CG, Calcium Green™-1 AM; OT, oxytocin.

Interestingly, the PLC inhibitor U73122 was able to prevent the facilitatory effect of OT 30 nM on both resting (Figure 4A,B) and 4-AP-evoked Ca^2+^ signals (Figure 4C,D). Notably, the inhibition of the PLC pathway not only abolished the facilitatory effect of OT on the 4-AP-evoked signal but also unmasked the inhibitory OT effect on the Ca^2+^ signal due to 4-AP (Figure 4C,D).

The finding suggests that the inhibition of the PLC pathway could unmask an inhibitory effect of OT 30 nM, therefore suggesting that OT 30 nM is able to activate coupling of the receptor to both the intracellular pathways, the inhibitory and the facilitatory.

### 3.3. Release of Glutamate from Astrocyte Processes: Insight into the Different Concentration-Dependent Responses to OT

In the attempt to better understand the divergent effects of OTR activation in the astrocyte processes, we checked if OTR coupling to different pathways might be involved. Therefore, we used pharmacological tools to modulate the pathways that had been coupled to OTR at neuronal levels and that were responsible for the inhibitory and the facilitatory effects of OT. In astrocyte processes, we assessed whether the Gi pathway, coupled to the inhibition of cAMP/PKA signaling [61,65,80,81], and the G_q_ pathway, coupled to the activation of PLC/PKC signaling [60,65], could affect the responses to OTR activation.

To interfere with the G_i_ inhibition of cAMP/PKA signaling, a PTX-sensitive mechanism [80,82], we checked the effects of PTX; to interfere with the G_q_ activation of PLC/PKC signaling, we checked the effects of the PLC inhibitor U73122 [83].

**Figure 5:**
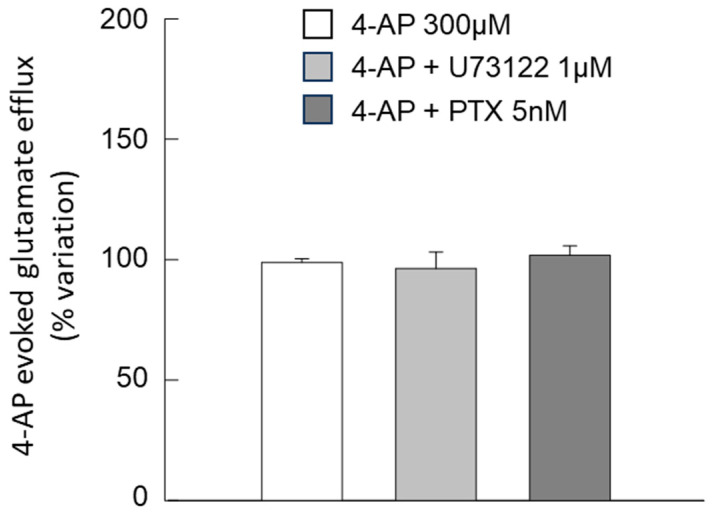
Endogenous glutamate release in response to 4-AP in striatal gliosomes. Ineffectiveness of PTX or the phospholipase PLC inhibitor U73122 of the 4-AP-evoked efflux. Briefly, gliosomes were exposed to 4-AP (6 min) during superfusion; PTX was entrapped at the used concentration during the tissue homogenization while U73122 was added 8 min before 4-AP. Additional details are reported in Section 2. Data are expressed as mean ± SEM of *n* = 6–23 independent experiments. One-way ANOVA analysis was applied to evaluate the effects of U73122 and PTX on 4-AP-evoked glutamate release (*p* = 0.6606; F (25) = 0.422). 4-AP, 4-aminopyridine; PTX, Pertussis Toxin.

Preliminary experiments showed that treatment with neither PTX (5 nM) nor U73122 (1 μM) had an effect per se on the 4-AP-evoked glutamate release (Figure 5).

**Figure 6:**
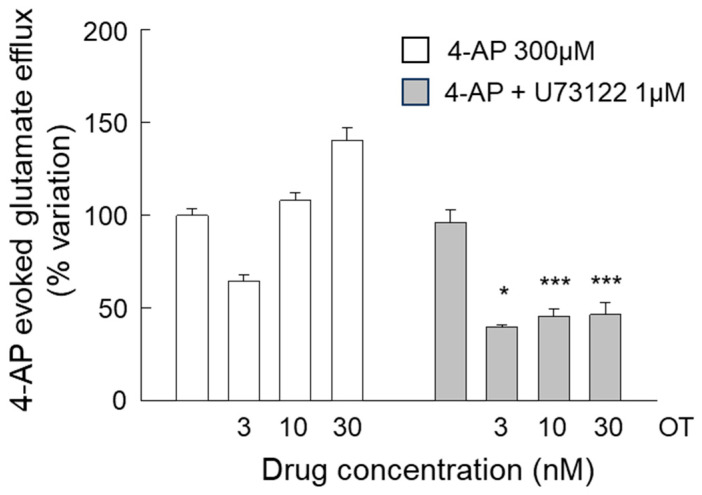
Endogenous glutamate release in response to 4-AP in striatal gliosomes. Effects of the PLC inhibitor U73122 on the OT (3–30 nM)-induced modification of the 4-AP-evoked efflux. Briefly, gliosomes were exposed to 4-AP (6 min) during superfusion; OT was added together with 4-AP; and U73122 was added 8 min before 4-AP. Additional details are reported in Section 2. Data are mean ± SEM of *n* = 5–8 independent experiments. Two-way ANOVA analysis was applied to evaluate the effects of U73122 at the different OT concentrations in 4-AP condition (*p* < 0.0001; F (38) = 38.51). * *p* < 0.05 and *** *p* < 0.01 compared with the effect of 4-AP + OT at the same concentrations but in the absence of U73122, according to two-way ANOVA plus Bonferroni’s multiple comparisons test. 4-AP, 4-aminopyridine; OT, oxytocin.

When the effect of the phospholipase PLC inhibitor U73122 was evaluated on the responses of the 4-AP-evoked efflux to OT (3–30 nM), surprisingly, but consistent with the finding on the Ca^2+^ signals, we found that the response to OT was reduced at all the concentrations that were tested (Figure 6). Moreover, as expected, the PLC inhibitor did not significantly modify the 4-AP-evoked calcium influx (see Figure S1). The findings indicate that OTR is capable of coupling to the facilitatory PLC pathway at all the OT concentrations tested (3, 10, and 30 nM).

**Figure 7:**
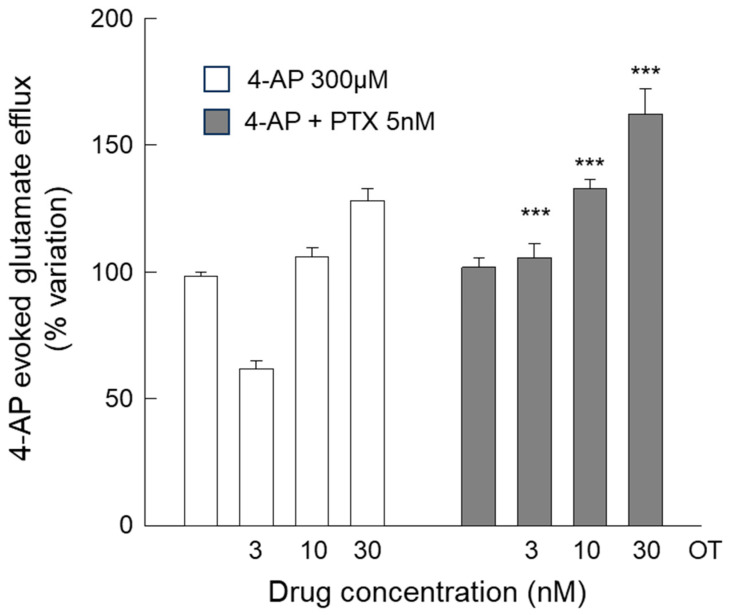
Endogenous glutamate release in response to 4-AP induced in striatal gliosomes. Effects of PTX on the OT (3–30 nM)-induced modification of the 4-AP-evoked efflux. Briefly, gliosomes were exposed for 6 min to 4-AP during superfusion; OT was added together with 4-AP; and PTX was added during gliosome preparation. Additional details are reported in Section 2. Data are mean ± SEM of *n* = 5–15 independent experiments. Two-way ANOVA analysis was applied to evaluate the effects of PTX at the different OT concentrations in the 4-AP condition (*p* < 0.0001; F (54) = 9.89). *** *p* < 0.01 compared with the effect of 4-AP + OT at the same concentrations but in the absence of PTX, according to the two-way ANOVA plus Bonferroni’s multiple comparisons test. 4-AP, 4-aminopyridine; OT, oxytocin; PTX, Pertussis Toxin.

Conversely, when the effect of the G_i_ inhibitor PTX was evaluated on the responses of the 4-AP-evoked efflux to OT (3–30 nM), we found that the OT response was modified at all the concentrations tested (Figure 7). The findings indicate that OTR is capable of coupling to the inhibition of the cAMP/PKA pathway at all the OT concentrations tested (3, 10, and 30 nM).

These findings corroborate the hypothesis that astrocytic OTR can be coupled to at least two pathways that have divergent effects on the glutamate release from the processes. The pathway coupled through a G_i_ to inhibition of cAMP/PKA signaling, leading to inhibition of the evoked glutamate release, and the pathway coupled to activation of PLC/PKC signaling, leading to facilitation of glutamate release, either in resting or stimulation conditions. OT appeared able to activate both the pathways, with the inhibitory response appearing only in stimulation conditions, while the facilitatory response appeared both in resting and in stimulated conditions. This was true for both glutamate release and Ca^2+^ signaling. Notably, the inhibitory and excitatory responses were evoked by the same tested OT concentrations, as indicated by the impact on the responses of the pharmacological manipulation of the pathways, namely of PTX inhibition of the G_i_ inhibitory pathway and of the selective inhibitor of the PLC/PKC pathway.

### 3.4. Release of Glutamate from Astrocyte Processes: Responses to OTR Biased Agonists

**Figure 8:**
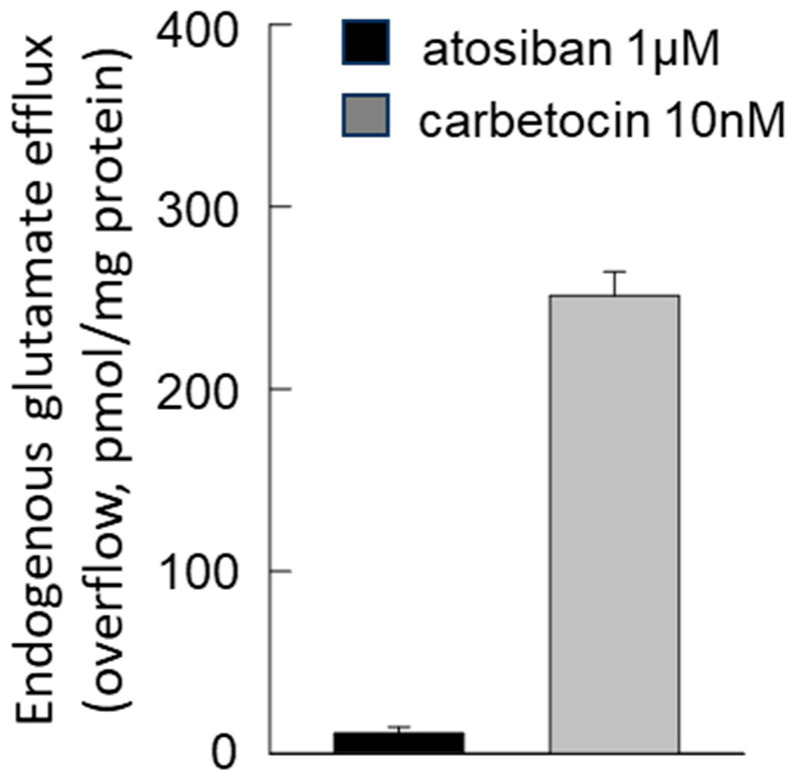
Endogenous glutamate release in response to atosiban and carbetocin in striatal gliosomes. Ineffectiveness of atosiban 1 μM and stimulation of the glutamate release by carbetocin 10 nM. Bars represent the overflow of the glutamate release, expressed as pmol/mg of protein, in the presence of atosiban and carbetocin at the concentrations indicated. Briefly, atosiban or carbetocin was added during superfusion (6 min). Additional details are reported in Section 2. Data are the mean ± SEM of *n* = 5 independent experiments.

Atosiban, the biased agonist reported to selectively promote OTR coupling to G_i_ [84], was unable to affect the resting glutamate release (Figure 8). Conversely, carbetocin, the biased agonist that selectively promotes OTR coupling to G_q_ [85], increased the resting transmitter efflux (Figure 8).

**Figure 9:**
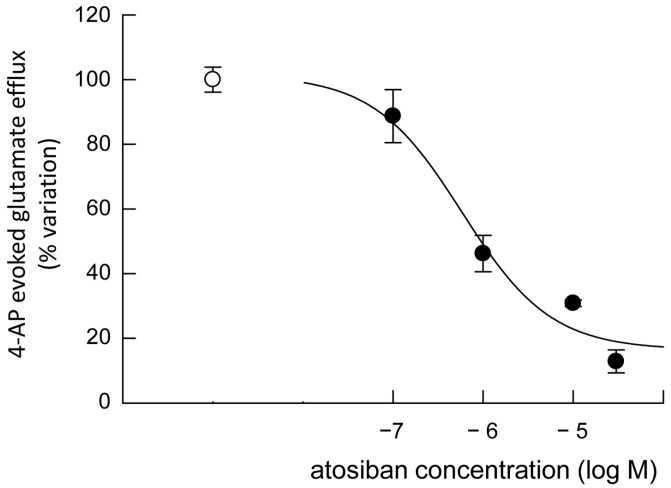
Concentration-response relationship of atosiban on the endogenous glutamate release in response to 4-AP induced depolarization in striatal gliosomes. 4-AP-evoked release (open symbol); percent variation in the 4-AP-evoked release in the presence of atosiban (filled symbols). Briefly, gliosomes were expoed for 6 min to 4-AP during superfusion; atosiban was added together with 4-AP. Additional details are reported in Materials and Methods section. Data are mean ± SEM of *n* = 5 independent experiments. 4-AP, 4-aminopyridine.

**Figure 10:**
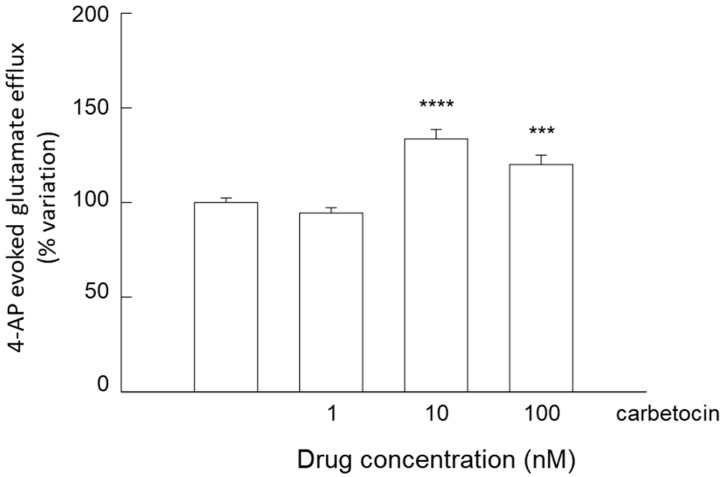
Effect of carbetocin on the endogenous glutamate release in response to 4-AP induced depolarization in striatal gliosomes. Briefly, gliosomes were exposed for 6 min to 4-AP during superfusion; carbetocin was added together with 4-AP. Additional details are reported in Section 2. Data are mean ± SEM of *n* = 5 independent experiments. One-way ANOVA analysis was applied to evaluate the effects of carbetocin at different concentrations in the 4-AP condition (*p* < 0.0001; F (27) = 23.757). *** *p* < 0.01; **** *p* < 0.001 compared with the effect of 4-AP according to the one-way ANOVA plus Bonferroni’s test. 4-AP, 4-aminopyridine.

On the other hand, studying the glutamate release evoked by 4-AP depolarization, atosiban inhibited the efflux in a concentration-dependent manner (Figure 9), whereas carbetocin increased it (Figure 10).

### 3.5. Ca^2+^ Signals in Astrocyte Processes: Responses to OTR Biased Agonists

**Figure 11:**
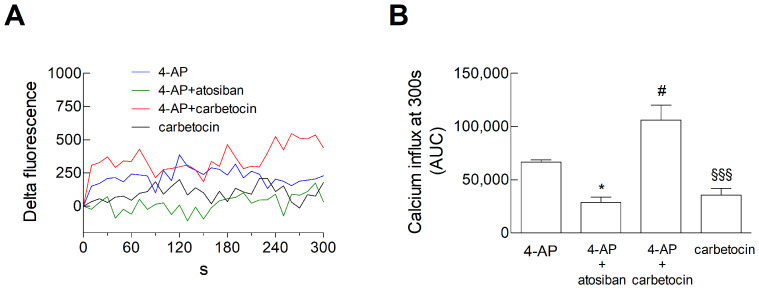
Calcium influx in response to 4-AP in striatal gliosomes. Gliosomes were loaded with CG and exposed to the indicated stimuli for 300 s at 37 °C. (**A**,**B**), and fluorescence related to CG was assessed every 10 s from 0 to 300 s. We expressed “Delta Fluorescence” as the [Ca^2+^]_i_ increase. Lines represent the mean values from *n* = 5 independent experiments, except “carbetocin” where *n* = 4 (**A**). The Ca^2+^ influx after 300 s was estimated by calculating the Areas Underlying the Curves (AUCs) and is reported in B for each experimental condition. Data are means ± SEM of *n* = 5 or 4 independent experiments. * *p* < 0.05 and # *p* < 0.05 compared with the effect of 4-AP, while §§§ *p* < 0.001 compared with the effect of 4-AP in the presence of carbetocin, according to one-way ANOVA (*p* < 0.001 and F (18) = 17.47), followed by Bonferroni’s post hoc test (**B**). 4-AP, 4-aminopyridine; CG, Calcium Green™-1 AM.

Consistent with the results obtained with OT, we have shown that carbetocin per se was able to increase the basal intracellular Ca^2+^ levels and Ca^2+^ levels evoked by 4-AP in striatal processes. Atosiban, on the other hand, could inhibit the Ca^2+^ levels evoked by 4-AP (Figure 11).

Therefore, the Ca^2+^ signal response to biased OT agonists appears to be consistent with the effects of these biased agonists on glutamate release.

## 4. Discussion

The functional evidence reported here was collected in freshly isolated astrocyte processes (gliosomes) from astrocytes in adult rat striata. We previously reported that our gliosomal preparation is a purified preparation of astrocyte processes, positive for the astrocytic marker GFAP and for ezrin, a marker of fine PAPs, and negative for neuronal markers, not contaminated by microglial or particles [72,73,86]. Notably, gliosomes were re-sealed subcellular particles obtained from branches of astrocytes that had maturated within striatal neuron–astrocyte networks in adult rats. Therefore, the findings reported here are likely to reflect the effects of OT on PAPs at tripartite glutamatergic synapses in adult rat striatum.

We previously reported that adult rat striatal astrocytes express OTR, and OTR activation on astrocyte processes could regulate the release of the gliotransmitter glutamate and Ca^2+^ signals in these processes [32,33]. The evidence here collected indicates that (1) OT can induce dual responses in astrocyte processes, namely the inhibition and facilitation of both Ca^2+^ signals and glutamate release; (2) the inhibitory response was dependent on the activation of a G_i_-dependent pathway; the facilitatory response was dependent on the activation of a PLC intracellular pathway; (3) both inhibitory and facilitatory responses were evoked by the same nanomolar OT concentrations; and (4) the biased agonists atosiban and carbetocin duplicated OT’s inhibitory and facilitatory effects, respectively, on both glutamate release and Ca^2+^ signals.

### 4.1. OT Can Induce Dual Responses in Astrocyte Processes, Namely the Inhibition and Facilitation of Both Ca^2+^ Signals and Glutamate Release

Dual responses, namely the inhibition and facilitation of the release of the gliotransmitter glutamate and of the Ca^2+^ signals, were dependent on OT activation of OTRs in the striatal astrocyte processes. The inhibitory response of glutamate (and of Ca^2+^ signals; see our previous data in [32]) was observed only when membrane was depolarized, while the facilitatory responses of glutamate and of Ca^2+^ signals appeared both in resting conditions and when depolarization was applied. Notably, OT effects on the Ca^2+^ signals duplicated the effects on glutamate release. Indeed, we already found that inhibition of the 4-AP-evoked Ca^2+^ signals in the astrocyte processes by 3 nM OT was paralleled by the inhibition of 4-AP-evoked release, consistent with OT’s ability to regulate the Ca^2+^-dependent exocytotic release of glutamate. In fact, we reported that 4-AP evoked a Ca^2+^-dependent exocytotic vesicular release of glutamate from the striatal astrocyte process [71], consistent with the ability of striatal astrocytes to release the gliotransmitter glutamate in situ in response to an increase in [Ca^2+^]_i_ [15] and with ultrastructural evidence for the presence of vesicular glutamate transporters in the process [72,73,74] as well as in striatal astrocytes in situ [87]. The ability of OT 30 nM to induce Ca^2+^ signals and glutamate release in these processes further supports the idea of OT regulation of Ca^2+^-dependent, exocytotic glutamate release. OT therefore appeared capable of regulating, either inhibiting or facilitating, Ca^2+^ events at the astrocyte processes and thus exocytotic glutamate release.

These findings appear of interest when considering that while both inhibitory and excitatory responses [59] were reported in neurons following OTR activation, to our knowledge only excitatory Ca^2+^ signals were reported in cultured astrocytes following OTR activation (in amygdala astrocytes [52], as well as in cultured hypothalamic astrocytes [49,50]). We here report that both inhibitory and excitatory responses could be evoked by OTR activation in the processes of astrocytes, indicating that the membrane state (therefore the conditions of the process, depending on the convergence of multiple signals) can drive the response to OT. As a matter of fact, the ability of OT to evoke both inhibitory and excitatory Ca^2+^ signals in astrocyte processes may not be surprising, as astrocytic Ca^2+^ signals may differ in different subcellular regions, with localized Ca^2+^ transients in fine astrocyte processes (the so-called microdomain Ca^2+^ events [88]) not reflecting somatic Ca^2+^ transients. Notably, microdomain Ca^2+^ events in astrocytic processes appear to be a key component of bidirectional astrocyte–neuron communication [89,90], playing an important role in the targeted release of gliotransmitters and local synaptic activity [91,92,93,94,95,96,97,98].

Furthermore, Ca^2+^ events can induce the remodeling of PAPs [99,100,101], changing synaptic coverage and therefore modulating gliotransmission and synaptic function [102]. In this context, it appears of interest to remember that OT seems to impact PAP motility and regulates synapse coverage [102,103,104,105]; see also [106]. The presence of OTR on striatal PAPs positive for ezrin (considering that ezrin is implicated in PAP motility and the regulation of synaptic coverage [107,108]) suggests that OT might control astrocytic coverage of striatal synapses. Therefore, OT, by regulating the diffusion of neurotransmitters to extrasynaptic targets [16,107,109,110], might contribute to balance and integrate wiring and volume transmission [44,111,112] in striatal integrative functions. By controlling glutamate release from the processes and balancing glutamate wiring and volume transmission, OT might possibly restore striatal glutamate transmission dysregulation related to astrocyte dysfunction in diseased conditions.

### 4.2. The Inhibitory Response Was Dependent on the Activation of G_i_ Coupled to the Inhibition of Adenyl Cyclase, and the Facilitatory Response Was Dependent on the Activation of the PLC Intracellular Pathway

It has been described that OTR can be coupled to both G_i_-dependent and G_q_-dependent pathways [66]. We here report that the excitatory response to OT of both glutamate release and Ca^2+^ signals was abolished by inhibiting receptor coupling to the G_q_-dependent PLC pathway. Conversely, the inhibitory response of glutamate release to OT was abolished by PTX inhibition of receptor coupling to the G_i_ pathway.

### 4.3. Both the Inhibitory and the Facilitatory Responses Were Evoked by OT in the Same Nanomolar Concentration Range

OT in the nanomolar range could activate both inhibitory and excitatory pathways. Specifically, OTR could be coupled to inhibitory or to both inhibitory and excitatory pathways depending on membrane depolarization. Notably, while no concentration dependency appeared for coupling to the inhibitory pathway in the range of 3–30 nM, coupling to the excitatory pathway was concentration-dependent in the nanomolar range. Consistently, selective inhibition of the PLC/PKC pathway reduced the response of OT 3–30 nM to the same level, unmasking the inhibitory effect of OTR activation in the range of 3–30 nM in depolarized conditions. On the other hand, PTX inhibition of coupling to the G_i_ pathway in depolarized conditions was able to increase the glutamate efflux evoked by OT in the concentration range of 3–30 nM, therefore unmasking the excitatory effect of OTR activation by OT 3–30 nM. As a matter of fact, the ineffectiveness of OT 10 nM was already reported on astrocytes in 4-AP-stimulated conditions [32] and confirmed here, appearing to be related to the activation of both the pathways, which masked the effect on each other.

It appears of special interest to note that the response to OT could be inhibitory, or both inhibitory and excitatory, depending on the membrane depolarization state; therefore, the membrane state of the astrocyte process, depending on the convergence of multiple signals can drive the response to OT. And indeed, astrocytes were reported to depolarize in response to neuronal activity, with the fluctuations of astrocytic membranes being closely related to neuron activity changes [113,114,115]. As far as the mechanisms involved in the depolarization-dependent appearance of an inhibitory response to OT, neuron activity-evoked depolarization of astrocytic membrane could activate cAMP synthesis [116], and cAMP was reported to increase the probability of astroglial vesicular secretion [117]. In this way, neuron activity can modulate the depolarization of astrocyte processes, which in turn regulates synaptic functioning through the release of gliotransmitters including glutamate.

It is worth noting that we report on the inhibitory or excitatory effects of OT in the low nanomolar range, reasonable for effective OTR concentrations in the brain (see [118]). It remains to be established if OT activation of striatal astrocytic inhibitory or excitatory pathways might be physiologically or pathologically relevant. Nevertheless, evidence that the (pharmacological or genetic) inactivation of OTRs in adult mice leads to abnormal striatal circuit functioning supports the role of OT and OTR activation for striatal circuit functioning, given that this inactivation might be behind the improper approach to novelty and the impaired processing of novel stimuli, which constitutes several aspects of autism spectrum disorder [36]. On the other hand, the therapeutic potential of OT in striatal disorders is suggested by the modulatory role of OT administration on social behavior in rodents [119] and on the functional connectivity at basal ganglia pathways that shape goal-directed behavior in humans [120]. Accumulating evidence on the involvement of striatal astrocytes in behavior regulation [121] suggests that striatal astrocytes might be involved in striatal dysfunction when OTRs are inactivated, as well as in OT therapeutic potential. The ability of OT to regulate the evoked glutamate release from striatal astrocyte processes adds complexity to the scene of OT’s modulatory effects in the brain’s complex integrative actions at tetrapartite synapses, formed by neural, glial, and extracellular molecular networks [122]. Due to the coupling of striatal astrocytic OTRs to different transduction pathways and the dual effects on Ca^2+^ signals and glutamate release, OT can exert a subtle regulation of striatal glutamatergic synapses, and OTRs may offer pharmacological targets at neuron–astrocyte networks to regulate glutamatergic striatal transmission that is potentially useful in striatal glutamatergic transmission dysregulation and in neuropsychiatric disorders. In fact, it is noteworthy that OT has been proposed as a promising treatment approach for PD [123,124]. More widely, the neuroprotective, anti-inflammatory, and antioxidant properties of OT leads us to propose OT administration to prevent neuron damage and death in neurodegenerative disorders [123,124,125,126,127]. As the role of alteration in astrocyte morphology and functioning is emerging in neurodegenerative/neurocognitive disorders (see [128,129,130,131,132], it can be proposed that astrocytic OT effects in striatum might support and amplify OT’s neuroprotective properties. This topic deserves further investigation.

### 4.4. The Biased Agonists Atosiban and Carbetocin Duplicate OT’s Inhibitory and Facilitatory Effects, Respectively, on Both Glutamate Release and Ca^2+^ Signals

Our observations indicated that OT directly engages and activates the G_q_ and G_i/o_ proteins at OTRs expressed on striatal astrocytic processes. These signaling pathways are involved in modulating intracellular Ca^2+^ signals and controlling the efflux of glutamate. The use of PTX clearly demonstrated the involvement of G_i/o_ proteins in response to different OT concentrations as U73122, the selective inhibitor of PLC, established the engagement of G_q_ proteins.

Peptide analogues of OT, synthetized by substituting specific key OT structural/functional residues, showed biased activation of G protein subtypes. Atosiban is an OT derivate. It has been considered a competitive antagonist on OTR/G_q_ coupling and only recently was suggested as a biased ligand for OTRs [84,133]. It displays agonist properties for its ability to activate G_i_ only, without the recruitment of β-arrestins, and then without inducing receptor internalization [65]. For all these characteristics, atosiban appears to show biased agonist properties. The effects of atosiban are here investigated to examine the specific contribution of the OTR-G_i_-coupled pathway in astrocytes.

Carbetocin is an OT-derived substance, synthesized to improve OT’s half-life and whose in vivo administration only partially mimics the effects of OT. In contrast to OT, and concerning the central effects, different (and in some cases opposite) effects have been reported. Similarly to OT, in an animal model, carbetocin reduced anxiety-like behaviors [134], had antidepressant-like effects [135], and attenuated the negative emotional consequences of opioid withdrawal [136,137]. By contrast, carbetocin did not have antipsychotic-like effects [138], slightly increased exploratory activity, and had no effects on grooming [139]. In a model of restraint stress, OT and carbetocin showed opposite effects on locomotion and grooming [140], and only carbetocin had long-term ameliorating effects on restraint stress-induced behavioral changes [141]. The differences observed between carbetocin and OT were explored at the molecular level in HEK293-transfected cells [85], suggesting that carbetocin has agonist properties on OTRs while being an antagonist on vasopressin receptors V1aR and V1bR. Activating OTR, carbetocin selectively engages the G_q_ pathway and promotes OTR internalization without inducing receptor recycling in the plasma membrane. To our knowledge, this is the first time that carbetocin was used on astrocytes and on astrocytic processes.

Atosiban mimicked OT’s inhibitory effects on both Ca^2+^ signals and glutamate efflux, with its effect appearing in depolarized conditions. On the other hand, carbetocin mimicked OT’s stimulatory effects on both Ca^2+^ signals and glutamate efflux, with its effect appearing both in resting and in depolarized conditions. This finding is consistent with the dual OT effect and coupling to different transduction pathways.

## 5. Conclusions

In conclusion, here we provided evidence for the ability of OTRs to regulate microdomain Ca^2+^ events and glutamate release in striatal astrocyte processes. In fact, OTRs could couple to both inhibitory and excitatory pathways, depending on the state of the depolarization of the membrane, and OT in a nanomolar range could activate both inhibitory and the excitatory pathways. We also addressed the molecular and intracellular pharmacology of OTRs and shed some light on the molecular cascade of events following OTR activation, revealing that both G_i_ and the G_q_/PLC pathway are activated following OTR activation in astrocyte processes. Although future studies are required to understand how OT affects astrocytic control of neuronal local circuity and synaptic activity, the knowledge presented in this study significantly advances our comprehension of OT’s effects on the brain. In fact, by modulating astrocytic Ca^2+^ signals and glutamate release and by regulating the signals impinging on synapses through PAP motility, including glutamate and OT itself, OT could play a role in bidirectional neuron–astrocyte communication and in the fine tuning of glutamatergic synapses. The present study was conducted on freshly prepared astrocytic processes. It may be important to replicate the investigation on OTR signaling in cultured astrocytes in future studies. While this study provides evidence that functional OTRs and dual responses to OT are characteristic of striatal PAPs in the control of glutamatergic transmission, the complexity of whole astrocytes and the integration of all signaling are neglected. However, the limitation of the biological model used in this study could also be seen as a strength, given that astrocytic processes are isolated from mature astrocytes in their physiological context. Although relevant issues remain to be addressed to understand changes in PAPs in the neuron–glia network in striatal neurodegenerative conditions, the observations suggesting dysregulation of striatal astrocytic control of glutamatergic transmission in PD are of significant interest, considering the regulation of glutamate release from the striatal PAPs by OTRs. As the role of alteration in astrocyte morphology and functioning is being established in neurodegenerative/neurocognitive disorders, it can be hypothesized that astrocytic OT effects in the striatum might support and amplify OT’s neuroprotective properties.

## Data Availability

Data are available on request from the corresponding authors.

## Supplementary Materials

The following supporting information can be downloaded at: https://www.mdpi.com/article/10.3390/biom15081122/s1, Figure S1: Effect of the phospholipase C inhibitor U73122 on 4-AP-evoked calcium influx.

## Abbreviations

4-AP	4-aminopyridine
cAMP	cyclic adenosine monophosphate
CG	calcium green
D2	dopamine D2 receptor
IP3	inositol-1,4,5-triphosphate
OT	oxytocin
OTR	oxytocin receptor
PAPs	perisynaptic astrocyte processes
PD	Parkinson’s disease
PKA	protein kinase A
PLC	phospholipase C
PTX	pertussis toxin
V1aR	vasopressin receptor 1a
V1bR	vasopressin receptor 1b
VGLUT	vesicular glutamate transporter
