# Supplementary material for: Dual Oxytocin Signals in Striatal Astrocytes
Source: Biomolecules. 2025 Aug 4;15(8):1122. doi: 10.3390/biom15081122 (PMC12383634; doi:10.3390/biom15081122)
Supplement: Supplementary file 1 [file biomolecules-15-01122-s001.zip › biomolecules-3752438-supplementary.pdf]

Article

Dual Oxytocin Signals in Striatal Astrocytes

Elisa Farsetti [1], Sarah Amato [1,†], Monica Averna [2], Diego Guidolin [3], Marco Pedrazzi [2], Guido Maura [4], Luigi Francesco Agnati [5], Chiara Cervetto [1,6,7,*] and Manuela Marcoli [4,7,*]

1 Department of Pharmacy, University of Genova, Viale Cembrano 4, 16148 Genova, Italy
2 Department of Experimental Medicine, University of Genova, Viale Benedetto XV 1, 16132 Genova, Italy
3 Department of Neuroscience, University of Padova, Via Gabelli 63, 35122 Padova, Italy
4 Department of Earth, Environment and Life Sciences, University of Genova, Viale Benedetto XV 5, 16132 Genova, Italy
5 Department of Biomedical, Metabolic Sciences and Neuroscience, University of Modena and Reggio Emilia, 41121 Modena, Italy
6 IRCCS Ospedale Policlinico San Martino, 16132 Genova, Italy
7 Interuniversity Center for the Promotion of the 3Rs Principles in Teaching and Research (Centro 3R), 56122 Pisa, Italy
* Correspondence: chiara.cervetto@unige.it (C.C.); manuela.marcoli@unige.it (M.M.)
† Current address: Institute of Neuroscience, National Research Council, Via Raoul Follereau 3, 20854 Vedano al Lambro (MB), Italy.

Supplementary Figure

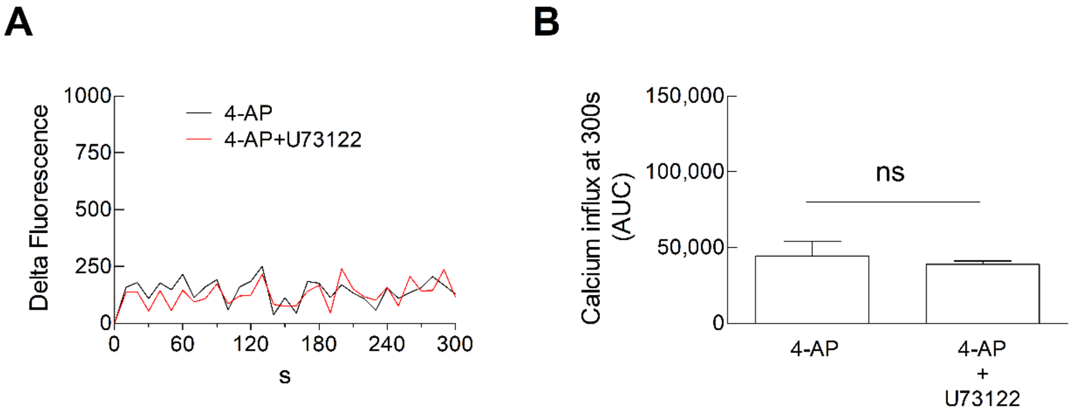

Figure S1. Effect of the phospholipase C inhibitor U73122 on 4-AP-evoked calcium influx. CG-loaded gliosomes were exposed to the indicated stimuli for 300 s at 37 °C. (A-B), CG-dependent fluorescence was monitored every 10 s from 0 to 300 s. $[Ca^{2+}]_i$ increase is expressed as "Delta Fluorescence". Lines represent the mean values from 4 independent experiments (A). The Ca^{2+} influx after 300 s was estimated by calculating the areas underlying the curves (AUC) and is reported in (B) for each experimental condition. Data are means ± SEM of 4 independent experiments. The difference between means was evaluated by two-tailed test ($p = 0.5864$, $t = 0.5746$); ns, (statistically) not significant. 4-AP, 4-aminopyridine; CG, Calcium Green™-1 AM.
